# Beyond Actionability: A Real-World Audit and Quality Improvement Initiative to Refine Shared Decision-Making Following 50-Gene Next-Generation Sequencing Results in Non-small Cell Lung Cancer

**DOI:** 10.7759/cureus.115487

**Published:** 2026-08-31

**Authors:** Arun Krishnan M.P., Vyshakh Thovarayi, Praveen K Shenoy, Nandini Devi R., Deepak Roshan

**Affiliations:** 1 Division of Medical Oncology, Malabar Cancer Centre – Post Graduate Institute of Oncology Sciences and Research, Thalassery, IND; 2 Division of Genetics, Malabar Cancer Centre – Post Graduate Institute of Oncology Sciences and Research, Thalassery, IND

**Keywords:** lung cancer, medical oncology clinic, next-generation sequencing, precision oncology, shared decision-making, targeted therapy

## Abstract

Background: Broad-panel next-generation sequencing (NGS) improves detection of clinically relevant genomic alterations beyond those detected by conventional targeted panels in advanced non-small cell lung cancer (NSCLC). However, the influence of these additional findings on shared decision-making (SDM) in routine clinical practice remains poorly described. This study evaluated the incremental value of an institutional 50-gene NGS panel in documenting SDM and characterized the genomic landscape in advanced NSCLC. Materials and methods: This retrospective observational study included consecutive patients with biopsy-proven advanced NSCLC who underwent in-house 50-gene NGS between June 2025 and June 2026 and had at least one documented post-NGS oncology consultation. Formalin-fixed paraffin-embedded tumor samples with ≥20% tumor cellularity were analyzed using an in-house 50-gene panel. Genomic alterations across all 50 genes were analyzed to characterize the molecular landscape, and alterations in the 37 genes exclusive to the expanded panel were evaluated for their impact on SDM. SDM was assessed across six predefined domains: targeted therapy discussion, molecular tumor board (MTB) referral, departmental discussion, clinical trial discussion, prognostic counseling, and genetic clinic referral. Results: A total of 113 patients were included (median age, 64.5 years; 74.3% males). The most frequent alterations in the 50-gene panel were TP53 (45.1%), KRAS (25.7%), EGFR (20.4%), CDKN2A (16.8%), FGFR3 (9.7%), and FGFR1 (7.1%). Additional genomic alterations exclusive to the expanded panel were identified in 49 patients (43.4%). However, only 16 patients (32.7%) had documented SDM influenced by these additional findings. Departmental discussion was the most frequently documented SDM domain (24.5%), whereas MTB referral (4.1%), targeted therapy discussion (4.1%), and clinical trial discussion (6.1%) were infrequent. No documented prognostic counseling or genetic referral was identified. A structured post-NGS SDM documentation template was developed.

Conclusions: Genomic profiling using a 50-gene NGS panel identified additional genomic alterations in patients with advanced NSCLC; however, documentation of the influence of these findings on SDM remained limited. Implementation of a standardized post-NGS documentation template may improve SDM documentation and facilitate more comprehensive integration of genomic findings into routine precision oncology practice.

## Introduction

The management of non-small cell lung cancer (NSCLC) has undergone a paradigm shift with the integration of molecular profiling and targeted therapies for actionable genomic alterations [1].

Initially, molecular testing in NSCLC was limited to identifying established driver alterations, such as EGFR, ALK, and ROS1, using PCR and FISH [2]. Subsequently, next-generation sequencing (NGS) enabled the simultaneous detection of multiple cancer-associated genes. The largest study from India on multi-gene NGS profiling by Sharma et al. used a customized 13-gene NGS panel. It concluded that multi-gene profiling improves the detection of actionable alterations and enables targeted therapies [3].

It is well established that using larger gene panels improves detection rates of genomic alterations and enhances precision oncology approaches. The ProfiLER-02 randomized trial reported a 14.8 percentage-point increase in the proportion of patients for whom a molecular-based recommended therapy was identified with comprehensive broad-panel profiling compared with a limited gene panel (51.6% vs. 36.9%, p < 0.001) [4].

Shared decision-making (SDM) following NGS testing is a collaborative process in which clinicians and patients discuss the genomic findings, their implications for treatment options, prognosis, eligibility for clinical trials, and future management strategies, while incorporating the patient's values and preferences to arrive at an individualized treatment plan. In broad panels, in addition to identifying established therapeutic targets, alterations in tumor suppressor genes and emerging biomarkers that may influence prognosis, eligibility for clinical trials, or future therapeutic strategies are also tested. Despite the increasing adoption of broad-panel NGS, the clinical significance of many additional genomic alterations and their translation into improved clinical outcomes remains uncertain [5,6]. Although these additional alterations detected by a large panel may not immediately alter systemic therapy, they can influence various aspects of treatment planning. These include triggering a molecular tumor board (MTB) discussion, consideration of off-label therapies, providing prognostic information, and the need for genetic evaluation. Consequently, these aspects influence the post-NGS SDM.

As many of the additional alterations may inform future treatment strategies, fully utilizing the benefits of the broad panel requires proper documentation of SDM. The transition from targeted panels to broad panels is happening slowly in low- and middle-income countries due to limited access, financial constraints, and a lack of expertise. SDM is minimally documented beyond actionability. Consequently, clinicians' post-large-panel NGS shared decision documentation is an evolving process that goes beyond identifying actionable targets. The documentation gaps need to be identified and addressed to better utilize the NGS studies.

Our institution transitioned from an outsourced 13-gene targeted NGS panel to an in-house 50-gene NGS panel for routine molecular profiling of lung cancer in June 2025. The expanded panel included the genes assessed by the previous targeted panel, along with additional genes, enabling characterization of incremental genomic findings identified through broader profiling. In this retrospective audit, we primarily evaluated whether additional genomic findings were documented as influencing predefined domains of post-NGS SDM. We additionally described the genomic landscape identified by the 50-gene panel and developed a structured post-NGS documentation template to address documented gaps as part of a quality-improvement initiative.

## Materials and methods

Study design and setting

This retrospective observational study was conducted in the Department of Clinical Hematology and Medical Oncology at Malabar Cancer Center, Kerala, India.

Study population

Consecutive patients with histologically confirmed advanced NSCLC who underwent molecular testing using the institutional 50-gene NGS panel between June 2025 and June 2026 were screened for eligibility. Patients were eligible for inclusion if they had histologically confirmed advanced NSCLC, underwent molecular profiling using the institutional in-house 50-gene NGS panel, and had at least one documented post-NGS consultation in the Department of Medical Oncology after the NGS report became available. Patients were excluded if they had inconclusive or failed NGS reports or incomplete or missing medical records that precluded assessment of post-NGS SDM documentation. Histological subtype was determined from the available pathology report. The cohort comprised all eligible patients seen consecutively during the predefined study period; no formal sample size calculation was performed because the study was a retrospective descriptive audit based on the available clinical cohort.

Molecular analysis

Tumor samples were analyzed using an in-house targeted 50-gene NGS panel designed to detect clinically relevant single-nucleotide variants (SNVs), insertions/deletions (indels), copy number alterations (CNAs), gene amplifications, and selected gene fusions. NGS was performed on formalin-fixed, paraffin-embedded tumor tissue specimens. Hematoxylin and eosin-stained sections were reviewed by pathologists, and only samples with a tumor cell content of ≥20% were selected for analysis. Genomic DNA was extracted using the NucleoSpin® DNA nucleic acid isolation kit (Macherey-Nagel GmbH & Co. KG, Düren, North Rhine-Westphalia, Germany), according to the manufacturer's instructions. DNA concentration was measured using the Qubit™ Fluorometer (Thermo Fisher Scientific, Waltham, Massachusetts, USA). Targeted NGS was performed using the Oncomine™ Precision Assay GX (Catalog No. A46291; Thermo Fisher Scientific, Waltham, Massachusetts, USA), a targeted 50-gene oncology panel designed for the detection of DNA sequence variants, copy number variations, and gene fusions on the Genexus™ Integrated Sequencer (Thermo Fisher Scientific, Waltham, Massachusetts, USA). Sequence data were analyzed using Genexus™ software (version 6.8.2), and clinically relevant variants were manually reviewed. Variant interpretation was performed using Oncomine™ Reporter (Thermo Fisher Scientific, Waltham, Massachusetts, USA) in accordance with the Association for Molecular Pathology (AMP)/American Society of Clinical Oncology (ASCO)/College of American Pathologists (CAP) guidelines [7]. The complete list of genes included in the 50-gene NGS panel is provided in the operational definition section.

Operational definition

The in-house 50-gene NGS panel included genes assessed for DNA sequence variants (SNVs/insertions and deletions (indels)), CNAs, and gene fusions. Genes evaluated for SNVs/indels were AKT1, AKT2, AKT3, ALK, AR, ARAF, BRAF, CDK4, CDKN2A, CHEK2, CTNNB1, EGFR, ERBB2, ERBB3, ERBB4, ESR1, FGFR1, FGFR2, FGFR3, FGFR4, FLT3, GNA11, GNAQ, GNAS, HRAS, IDH1, IDH2, KIT, KRAS, MAP2K1, MAP2K2, MET, MTOR, NRAS, NTRK1, NTRK2, NTRK3, PDGFRA, PIK3CA, PTEN, RAF1, RET, ROS1, SMO, and TP53; genes assessed for CNAs were ALK, AR, CD274 (PD-L1), CDKN2A, EGFR, ERBB2, ERBB3, FGFR1, FGFR2, FGFR3, KRAS, MET, PIK3CA, and PTEN; and genes assessed for fusions were ALK, AR, BRAF, EGFR, ESR1, FGFR1, FGFR2, FGFR3, MET, NRG1, NTRK1, NTRK2, NTRK3, NUTM1, RET, ROS1, RSPO2, and RSPO3.

Additional genomic alterations definition

Additional genomic alterations were defined as pathogenic or likely pathogenic genomic alterations detected in any of the 37 genes exclusive to the institutional 50-gene NGS panel. Alterations in the 13 genes common to both the outsourced 13-gene panel and in-house 50-gene panels (EGFR, ALK, ERBB2, BRAF, KRAS, MAP2K1, NRAS, PIK3CA, MET, RET, ROS1, TP53, NTRK1, NTRK2, and NTRK3) were excluded from this definition. The terms "additional genomic alteration," "additional variant," "additional genomic finding," "additional gene finding," and "additional 37-gene alteration" are used synonymously throughout this manuscript.

SDM impact definition as a composite outcome

The primary outcome was the impact on SDM attributable to additional genomic alterations identified by the in-house 50-gene panel. SDM impact was defined as documentation of one or more of the following six domains during the post-NGS medical oncology consultation: (1) targeted therapy discussion, defined as documentation of consideration or intent to initiate targeted therapy based on the additional genomic alteration; (2) MTB discussion, defined as documentation that the case was referred or planned for MTB review because of the additional genomic alteration; (3) departmental discussion, defined as documentation of consultant-level or multidisciplinary departmental discussion influenced by the additional genomic alteration; (4) clinical trial discussion, defined as documentation of counseling regarding eligibility for a clinical trial based on the additional genomic alteration; (5) prognostic discussion, defined as documentation explicitly linking the additional genomic alteration to prognosis or prognostic clarification; and (6) genetic clinic referral, defined as documentation of referral for genetic counseling or clinical genetics evaluation based on the additional genomic alteration.

Composite outcome of SDM impact

SDM was considered present (yes) if at least one of the six SDM domains was documented and absent (no) if none of the six SDM domains was documented.

Study objectives

The primary objective was to estimate the proportion of patients with additional genomic alterations identified by the expanded 50-gene NGS panel who had documented SDM impact attributable to these findings. The secondary objectives were to describe the genomic landscape identified by the institutional 50-gene NGS panel and to develop a structured documentation template addressing gaps in post-NGS SDM documentation.

Quantifying the documented SDM impact was undertaken as a quality-improvement measure to identify gaps in the translation of additional genomic findings into clinical documentation. Characterization of the genomic landscape provided context for the incremental findings identified through expanded molecular profiling.

Data collection and statistical analysis

Data were extracted retrospectively from institutional medical records and NGS reports using a predefined data collection pro forma. Demographic characteristics and genomic alterations identified by the 50-gene panel were collected. Documentation of post-NGS consultations was reviewed for the predefined SDM domains. To evaluate the impact on SDM, alterations involving the 13 genes common to the conventional panel were excluded. The retrospective extraction was performed by the first author through review of the available clinical records and NGS reports.

The primary endpoint was the proportion of patients with additional genomic alterations who had documented SDM impact attributable to these findings. The denominator for the primary SDM analysis was the 49 patients in whom at least one pathogenic or likely pathogenic alteration was identified in the 37 genes exclusive to the expanded panel. For the assessment of SDM documentation, only the first oncology consultation at which the NGS report was available was evaluated; subsequent consultations were not included in the primary analysis.

Descriptive statistics were used to summarize genomic alterations and SDM documentation. Categorical variables were presented as frequencies and percentages. No formal sample size calculation was performed because all eligible patients who were seen consecutively during the predefined study period were included. Missing variables were not imputed, and analyses were based on available documentation. No inferential subgroup analysis or multivariable modeling was performed because the study was designed as a descriptive retrospective audit and the number of patients with documented SDM impact was limited. Accordingly, no formal statistical significance threshold was applied. Statistical analyses were performed using SPSS Statistics version 29.0 (IBM Corp. Released 2022. IBM SPSS Statistics for Windows, Version 29.0. Armonk, NY).

Ethical considerations

This study was conducted after obtaining approval from the Institutional Review Board of Malabar Cancer Centre (approval number: 1616/IRB-SRC/13/MCC/23-05-2026/4, dated May 23, 2026). As the study was a retrospective audit of routinely collected clinical data, the IRB waived the requirement for informed consent.

## Results

A total of 113 patients with advanced NSCLC who underwent in-house 50-gene NGS and had at least one documented post-NGS medical oncology clinic consultation were included in the analysis. The median age was 64.5 years (range, 37-87 years). Among the 113 patients, 84 (74.3%) were male. Adenocarcinoma was the most frequent histological subtype (63, 56.6%), followed by squamous cell carcinoma (34, 30.0%) and NSCLC not otherwise specified (16, 14.2%). The frequency of genomic alterations in the 13 clinically established genes included in the institutional 50-gene NGS panel is summarized in Table 1.

**Table 1:** Frequency of genomic alterations in the 13 clinically established genes within the institutional 50-gene NGS panel (N = 113) This table summarizes the frequency of pathogenic or likely pathogenic genomic alterations identified in the 13 clinically established genes routinely evaluated for therapeutic decision-making in advanced NSCLC. All 113 patients underwent molecular profiling using the institutional in-house 50-gene NGS panel. Data are presented as the number of patients with at least one alteration in each gene (n (%)). NGS: next-generation sequencing, NSCLC: non-small cell lung cancer

Gene altered	N = 113 (%)
EGFR	23 (20.4)
ALK	8 (7.1)
ERBB2 (HER2)	6 (5.3)
BRAF	8 (7.1)
KRAS	29 (25.7)
MAP2K1	0 (0)
NRAS	0 (0)
PIK3CA	7 (6.2)
MET	5 (4.4)
RET	1 (0.9)
ROS1	4 (3.5)
TP53	51 (45.1)
NTRK 1, 2, and 3	0 (0)

The distribution of pathogenic or likely pathogenic genomic alterations identified in the 37 genes exclusive to the expanded 50-gene NGS panel is presented in Table 2.

**Table 2:** Frequency of genomic alterations in genes exclusive to the expanded 50-gene NGS panel (excluding the 13 clinically established genes) (N = 113) This table presents the distribution of pathogenic or likely pathogenic genomic alterations detected in the 37 additional genes included exclusively in the institutional 50-gene NGS panel. Alterations in the 13 clinically established genes common to both the conventional targeted panel and the expanded panel, EGFR, ALK, ERBB2 (HER2), BRAF, KRAS, MAP2K1, NRAS, PIK3CA, MET, RET, ROS1, TP53, and NTRK 1, 2, and 3, were excluded. Data are presented as the number of patients with each genomic alteration (n (%)), highlighting the additional molecular information obtained through 50-gene profiling beyond the conventional 13-gene targeted panel. CD274 alterations detected by NGS were considered genomic findings and were not interpreted as a substitute for clinically validated PD-L1 protein expression assessed by immunohistochemistry. NGS: next-generation sequencing

Gene altered	N = 113 (%)
CDKN2A	19 (16.8)
FGFR3	11 (9.7)
FGFR1	8 (7.1)
PTEN	5 (4.4)
HRAS	4 (3.5)
IDH2	2 (1.8)
ERBB3	2 (1.8)
CD274	2 (1.8)
CTNNB1	1 (0.9)
IDH1	1 (0.9)
KIT	1 (0.9)
FLT3	1 (0.9)
GNAS	1 (0.9)
CHEK2	1 (0.9)
MTOR	1 (0.9)
SMO	1 (0.9)
ESR1	1 (0.9)

Documentation gaps identified 

This audit identified substantial gaps in the documentation of SDM following 50-gene panel NGS in advanced NSCLC. Two distinct documentation gaps were noted: (1) failure to document the additional genomic finding in the case record and (2) failure to document its implications in SDM, including departmental discussion, targeted therapy discussion, clinical trial consideration, prognostic counseling, or genetic clinic referral.

Additional genomic alterations beyond the conventional 13-gene panel were identified in 49/113 patients (43.4%). Among these 49 patients with genomic alterations in the additional 37 genes, 34/49 (69.4%) had documentation of the identified variant in the post-NGS oncology consultation, whereas 15/49 (30.6%) had no documentation of the genomic finding in clinician notes despite its presence in the NGS report. Furthermore, only 16/49 patients (32.7%) had documented evidence that these findings influenced the domains of SDM. The remaining 33/49 (67.3%) had no documentation indicating that the additional genomic alteration was discussed in a manner that influenced clinical decision-making. The documented impact of additional genomic alterations identified exclusively by the expanded 50-gene NGS panel on SDM is illustrated in Figure 1.

**Figure 1:**
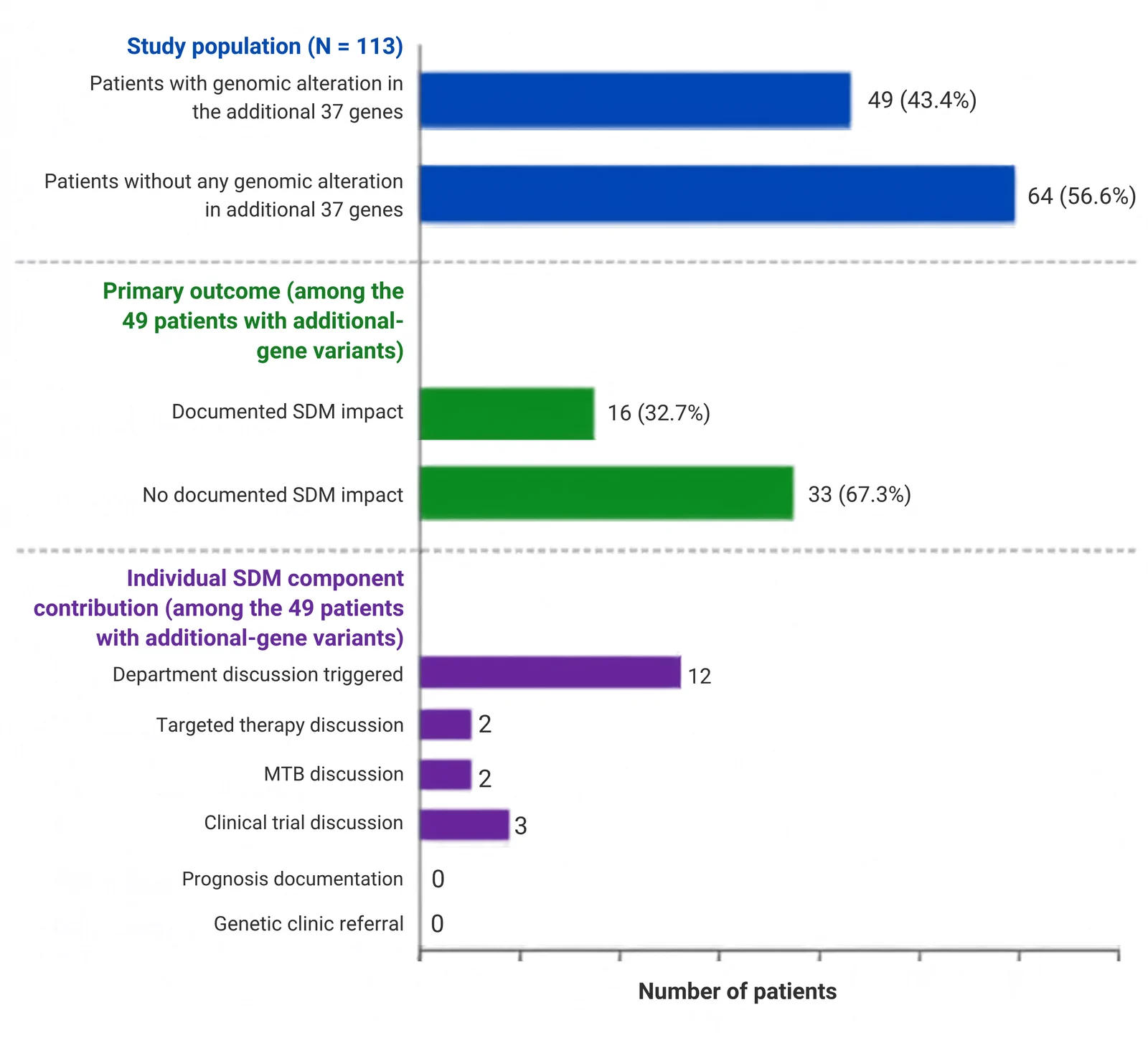
Impact of genomic alterations exclusive to the 50-gene panel on documented SDM This figure illustrates the frequency of documented SDM domains among the 49 patients with pathogenic or likely pathogenic genomic alterations detected exclusively in the additional 37 genes (excluding EGFR, ALK, ERBB2, BRAF, KRAS, MAP2K1, NRAS, PIK3CA, MET, RET, ROS1, TP53, and NTRK) of the institutional 50-gene NGS panel. Bars represent the number and percentage of patients in whom additional genomic findings led to departmental discussion, an MTB referral, consideration of targeted therapy, clinical trial discussion, prognostic counseling, or referral for genetic evaluation during the post-NGS oncology consultation. Data are presented as the number and percentage of patients (n (%)). Patients could contribute to more than one SDM domain; therefore, the categories are not mutually exclusive. SDM: shared decision-making, NGS: next-generation sequencing, MTB: molecular tumor board

The most commonly documented clinician response to additional findings was initiation of departmental discussion (12/49, 24.5%), whereas documentation of clinical trial discussion (3/49, 6.1%), targeted therapy consideration (2/49, 4.1%), and MTB referral (2/49, 4.1%) was infrequent. No patient record had documentation linking additional genomic findings to prognostic counseling or referral for genetic evaluation.

## Discussion

These findings demonstrate that although broad-panel NGS identified additional genomic alterations in nearly half of the study population, the translation of these findings into documented SDM was limited.

Genomic landscape in our study versus global and Indian studies

In this real-world cohort of 113 patients with advanced NSCLC, TP53 (51/113, 45.1%), KRAS (29/113, 25.7%), and EGFR (23/113, 20.4%) were the most frequently altered genes. The frequency of EGFR alterations was slightly lower than that reported in previous Indian studies, in which EGFR mutations were identified in approximately 23%-35% of NSCLC cases [8-10]. The prevalence of ALK and ROS1 alterations was broadly consistent with contemporary Indian genomic studies. In contrast, the frequency of KRAS alterations (25.7%) was higher than the 13.3% reported in the large Indian NGS cohort by Sharma et al. [3]. These differences in EGFR and KRAS frequencies may reflect variations in patient characteristics, including the higher proportion of male patients and greater representation of squamous cell carcinoma in our cohort, as well as differences in sequencing methodology.

TP53 remained the most common co-occurring alteration, consistent with Indian datasets [3,8]. In the whole cohort of 113 patients, among the genes exclusive to the 50-gene panel, CDKN2A (19/113, 16.8%) was the most frequent alteration, followed by FGFR3 (11/113, 9.7%) and FGFR1 (8/113, 7.1%), highlighting the broader molecular heterogeneity captured by expanded testing. While recent Indian broad-panel NGS studies have similarly identified recurrent alterations beyond the conventional driver genes, the distribution of individual additional gene alterations varies across cohorts [8,9].

Increased detection due to the 50-gene panel: beyond actionability

The transition from a 13-gene panel to a 50-gene panel identified additional genomic alterations in 49 patients (43.4%). While many of these alterations were not immediately actionable, they provided clinically relevant information on tumor biology, potential eligibility for clinical trials, future therapeutic strategies, and the need for multidisciplinary review. This observation aligns with studies such as ProfiLER-02, which demonstrated that broader genomic profiling increases the identification of potentially actionable or investigational molecular alterations beyond standard driver testing [4]. The ProfiLER-02 study evaluated the impact of broader molecular profiling on the identification of molecularly guided treatment opportunities. In contrast, our study evaluates the documentation of SDM following the detection of additional genomic alterations. Similar real-world studies have shown that comprehensive genomic profiling frequently influences treatment planning, even when it does not immediately lead to the initiation of targeted therapy. Instead, genomic findings often contribute to clinical trial enrolment, MTB recommendations, future therapeutic planning, and refinement of the overall management strategy [11,12].

Documentation gaps identified in the study and proposed template

These findings suggest a substantial gap in translating NGS results into structured clinical conversations and documenting those discussions. One possible reason for this gap is that, in routine practice, clinicians might have discussed the implications of these findings and trial opportunities verbally but failed to document them. Despite additional alterations being identified in nearly half the cohort, only one-third had any documented SDM impact. These gaps likely reflect the lack of a structured post-NGS counseling documentation framework rather than a complete absence of clinical conversations.

As institutions gradually transition from limited, targeted panels to broad panels, a standardized post-NGS documentation framework is needed. Such a framework can improve the quality of care delivered and serve as a checkpoint to prevent clinicians from missing critical domains of post-NGS counseling [13]. It ensures that the broader clinical value of NGS findings, beyond immediate actionability, is utilized appropriately. The absence of a structured documentation format or checklist also limits the ability to audit the quality of care and assess the real-world incremental value of expanding the gene panel. Taking these facts into consideration, we propose a template for documenting post-NGS counseling, as depicted in Table 3.

**Table 3:** Post-broad-panel NGS SDM template This table outlines the structured documentation template developed as part of a quality improvement initiative, based on the findings of the present audit. The template is intended to standardize documentation following broad-panel NGS testing by capturing the identified genomic alteration, key domains of SDM, including targeted therapy consideration, MTB referral, departmental discussion, clinical trial eligibility, prognostic counseling, and genetic counseling referral, as well as the final management plan, patient preference, and follow-up. The template is designed to facilitate comprehensive post-NGS counseling and improve the quality and consistency of clinical documentation. NGS: next-generation sequencing, SDM: shared decision-making, MTB: molecular tumor board

Patient ID: ______ Date: ______ Diagnosis: ______ Stage: ______
Genetic alteration identified: Yes/No Name the gene alteration detected: ______
SDM checklist
Targeted therapy considered based on ______ alteration: Yes/No
MTB referral planned based on ______ alteration: Yes/No
Departmental multidisciplinary discussion performed based on ______ alteration: Yes/No
Clinical trial eligibility discussed based on ______ alteration: Yes/No
Prognostic implications explained based on ______ alteration: Yes/No Genetic counseling referral considered based on ______ alteration: Yes/No
Final plan
Management decision after SDM: ______
Patient preference documented: Yes/No
Follow-up plan: ______

Strengths of the study

This study is among the first real-world Indian quality-improvement audits to evaluate the impact of 50-gene panel NGS on SDM and to report the genomic landscape. Strengths include the consecutive inclusion of patients, assessment of multiple SDM domains, and the development of a practical documentation template that can be implemented in routine oncology practice.

Limitations of the study

This study has several limitations. First, its retrospective single-center design and relatively modest sample size limit generalizability. Second, inclusion required at least one documented post-NGS oncology consultation; therefore, patients without post-NGS follow-up, including those with rapid clinical deterioration, loss to follow-up, or limited access to care, may have been excluded, introducing potential selection bias. Third, absence of documentation cannot be equated with absence of a clinical discussion, as verbal discussions may not have been recorded in the available clinical record. Fourth, the six SDM domains were combined into a composite outcome despite potentially differing clinical significance, and the study was not designed to identify independent predictors of SDM impact. Fifth, the institutional 50-gene panel and the clinical characteristics of this cohort may differ from those of other centers, limiting external generalizability. Sixth, the study period was relatively short and occurred early in the transition to the expanded panel, when clinician familiarity with the broader genomic findings may have been evolving. Seventh, the proposed documentation template has not yet undergone prospective validation, and its effect on documentation quality or clinical outcomes remains unknown. Finally, genomic actionability is evolving rapidly, and the distinction between actionable and non-actionable alterations may change as evidence and therapeutic options develop.

## Conclusions

Genomic profiling using a 50-gene NGS panel identified additional genomic alterations in patients with advanced NSCLC; however, documentation of the influence of these findings on SDM remained limited. Implementation of a standardized post-NGS documentation template may improve SDM documentation and facilitate more comprehensive integration of genomic findings into routine precision oncology practice.
